# Phase I study of adjuvant chemotherapy with nab‐paclitaxel and S‐1 for stage III Lauren's diffuse‐type gastric cancer after D2 resection (NORDICA study)

**DOI:** 10.1002/cam4.4966

**Published:** 2022-06-25

**Authors:** Yuehong Cui, Yiyi Yu, Shan Yu, Wei Li, Yan Wang, Qian Li, Tianshu Liu

**Affiliations:** ^1^ Medical Oncology Department of Zhongshan Hospital affiliated to Fudan University Shanghai China; ^2^ Cancer Center of Zhongshan Hospital Fudan University Shanghai China

**Keywords:** gastric cancer, maximal tolerated dose, nab‐paclitaxel, phase I, S‐1

## Abstract

**Purpose:**

The prognosis of diffuse‐type gastric cancer (DGC) is poorer than that of intestinal type, but S‐1 is a potential treatment option in DGC. This study explored the maximal tolerated dose (MTD) and the recommended dose for phase II study (RP2D) of nab‐paclitaxel combined with S‐1 (AS regimen) as adjuvant chemotherapy in stage III DGC.

**Methods:**

Patients with stage III DGC were recruited into this phase I dose‐escalation study between July 2019 and June 2020 in Zhongshan Hospital. Nab‐paclitaxel and S‐1 (80–120 mg/day, d1‐14, q3w) were administrated for 6 cycles, and then 8 cycles of S‐1 monotherapy were applied. The patients received nab‐paclitaxel at 180, 220, or 260 mg/m^2^ according to the 3 + 3 design based on dose‐limiting toxicity (DLT). The primary endpoint was RP2D. Secondary endpoints were the 1‐year disease‐free survival (DFS) rate and adverse events (AEs).

**Results:**

One case experienced DLT in 180‐mg/m^2^ dose group, subsequently three additional subjects were enrolled. DLT was not observed in the 220‐ and 260‐mg/m^2^ dose groups (both *n* = 3). Therefore, the MTD has not reached, and the RP2D of nab‐paclitaxel would be 260 mg/m^2^. Five participants showed progressive disease, with three and two participants in the 180‐ and 220‐mg/m^2^ dose groups, respectively. The 6‐, 12‐, and 18‐month DFS rates were 100%, 63.6%, and 50.9%, respectively. The most frequently observed AEs were neutropenia (83.3%) and leukopenia (66.7%).

**Conclusion:**

The RP2D of nab‐paclitaxel as adjuvant chemotherapy in DGC was 260 mg/m^2^. The AS regimen had a tolerable AE profile in stage III DGC.

## Graphical abstract

This phase I dose‐escalation study of adjuvant nab‐paclitaxel plus S‐1 (AS) recruited patients with stage III DGC. The RP2D of nab‐paclitaxel as adjuvant chemotherapy in DGC was 260 mg/m^2^. The AS regimen had a tolerable AE profile in stage III DGC.

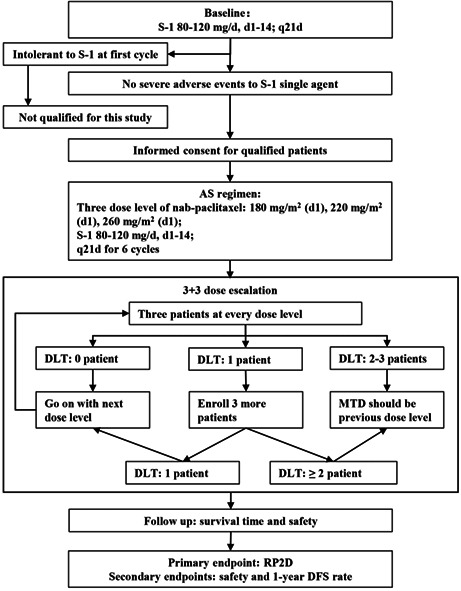

## 1 INTRODUCTION

Gastric cancer (GC) remains a prevalent malignant tumor in the world with a poor prognosis, which seriously threatens human health.^1^, ^2^ GC incidence is highest in Eastern Asia,^1^ with men affected more and^1^, ^2^, ^3^ suspected culprits of *Helicobacter pylori* infection and some hereditary cancer predisposition syndromes.^4^ In China, nearly 70% of gastric cancers are detected at an advanced stage.^5^, ^6^ Based on the Lauren classification, GC is classified into two types—intestinal type (IGC) and diffuse‐type GC (DGC).^3^, ^7^ The IGC principally includes well‐differentiated cancers, while DGC includes poorly differentiated cancers with a poorer prognosis,^3^, ^8^ and it is urgent to improve the prognosis of DGC. However, few studies evaluated the efficacy of adjuvant chemotherapy regimens in DGC only. Surgical resection is the major method for advanced GC, but most patients cannot be cured by surgery alone.^7^, ^9^ Therefore, postoperative adjuvant chemotherapy occupies a pivotal position in GC.

Adjuvant S‐1 monotherapy,^10^, ^11^ oxaliplatin plus capecitabine,^12^, ^13^ or docetaxel plus S‐1^14^ can delay the progression of gastric cancer compared with surgery alone. The authors' team analyzed 331 postoperative patients with DGC retrospectively, and demonstrated that the overall survival (OS) and disease‐free survival (DFS) in patients treated with oxaliplatin‐containing regimens were not different from those without oxaliplatin, suggesting that DGC patients might benefit less from oxaliplatin‐based adjuvant therapy than IGC.^15^ Besides, it is reported that taxanes plus fluoropyrimidine showed a better DFS and OS compared oxaliplatin plus fluoropyrimidine in the treatment of DGC after D2 gastrectomy.^16^, ^17^

Nukatsuka et al.^18^ confirmed the synergistic effects of S‐1 and paclitaxel in xenograft mouse models of breast cancer. Subsequent clinical studies showed that the objective response rates (ORR) could reach 50%–60% with such combination.^19^, ^20^, ^21^ Furthermore, as a novel type of paclitaxel, nab‐paclitaxel has shown favorable efficacy and fewer adverse events (AEs) in metastatic GC than solvent‐based paclitaxel.^22^, ^23^, ^24^, ^25^ Notably, preclinical data have shown that the combination of nab‐paclitaxel and S‐1 also has a synergistic effect on suppressing tumor progression in a mouse model.^26^ Although the safety and efficacy of nab‐paclitaxel in metastatic gastric cancer patients have been demonstrated in previous studies,^22^, ^23^, ^24^, ^25^ it is still necessary to explore the tolerability of nab‐paclitaxel in adjuvant chemotherapy.

Therefore, this phase I trial of adjuvant chemotherapy with nab‐paclitaxel and S‐1 (AS regimen) aimed to explore the maximal tolerated dose (MTD) and the recommended dose of phase II (RP2D) in patients with stage III DGC after D2 radical gastrectomy, and to preliminarily investigate the treatment effect and AEs of this regimen.

## 2 METHODS

### 2.1 Study design and participants

This phase I dose‐escalation study (NCT03977220, ClinicalTrials.gov) of adjuvant chemotherapy with nab‐paclitaxel and S‐1 recruited participants with stage III adenocarcinoma of the stomach or gastroesophageal junction (GEJ) of Lauren's diffuse type from July 2019 to June 2020 in Zhongshan Hospital. Detailed inclusive and exclusive criteria were provided in the Supplementary material.

The Ethics Committee of Zhongshan Hospital approved this trial (approval number: B2019‐127R). The informed consent for study participation was obtained from all subjects. The study was carried out based on the Declaration of Helsinki and the Good Clinical Practice.

### 2.2 Treatment

The participants received S‐1 monotherapy as the first cycle of adjuvant treatment. In the absence of grade ≥3 chemotherapy‐related AEs, nab‐paclitaxel and S‐1 which were based on a regimen of S‐1 combined with docetaxel^14^ were administered for the subsequent treatments. There were two reasons to exclude the patients who were intolerant to S‐1 monotherapy. First, considering the safety of patients who were intolerant to S‐1 monotherapy, combination chemotherapy regimen might cause serious adverse events. Second, due to the strong evidence of S‐1 in adjuvant therapy after gastric cancer radical resection, the purpose of this study is mainly to explore the RP2D of nab‐paclitaxel, we had to eliminate disturbance of S‐1.

**FIGURE 1:**
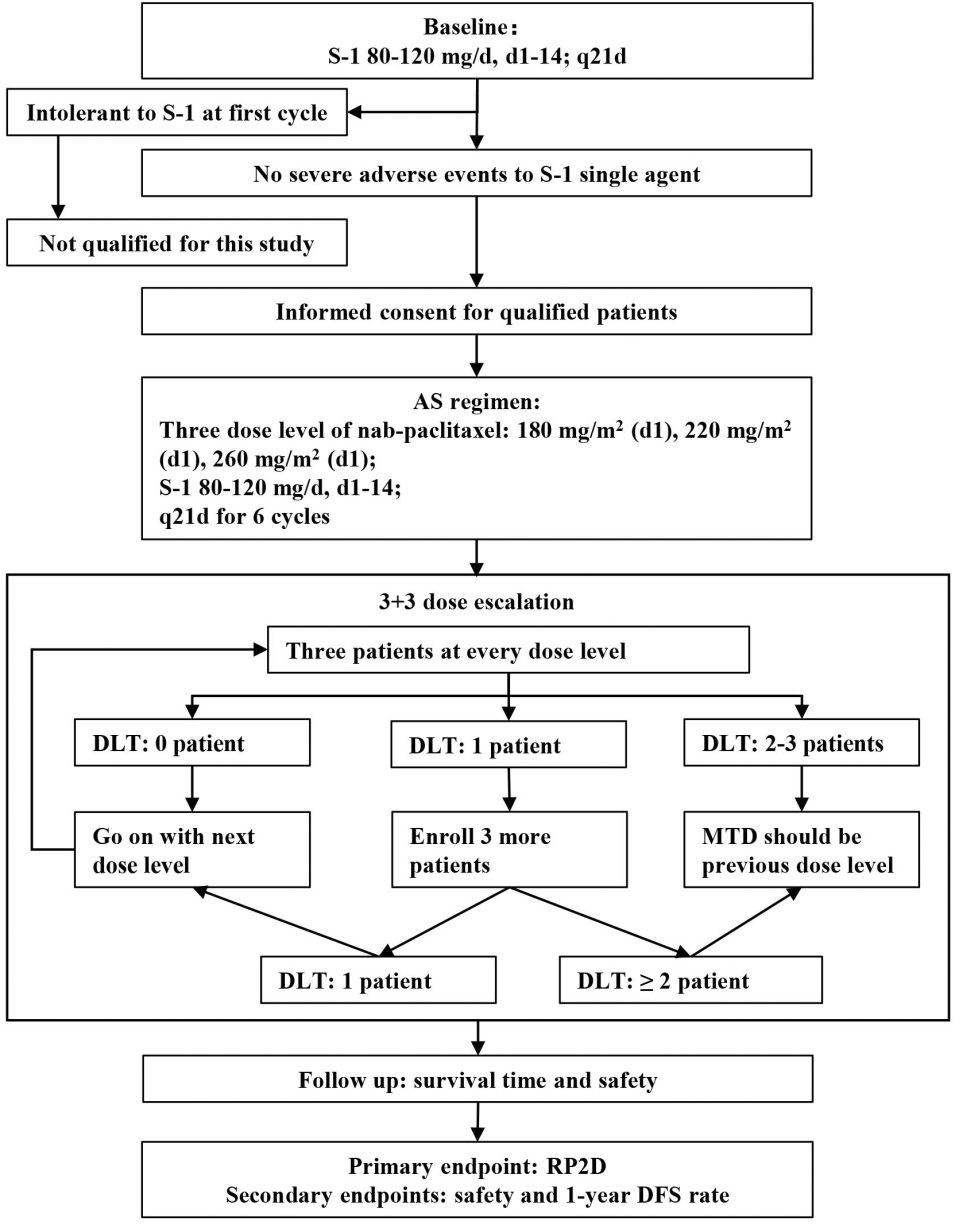
The flow diagram of this study. AS, nab‐paclitaxel combined with S‐1. q21d, repeat every 21 days; DLT, dose‐limiting toxicity; MTD, maximal tolerated dose; DFS, disease‐free survival.

Nab‐paclitaxel in a dose of 180, 220, or 260 mg/m^2^ ^25^ was administrated on day 1 of every 21 days. S‐1 was given orally twice a day at 80–120 mg/day based on body surface area (BSA) (80 mg/d for BSA <1.25 m^2^; 100 mg/day for 1.25 ≤ BSA <1.49 m^2^; 120 mg/day for BSA ≥1.5 m^2^) for 6 cycles (day 1 to 14, every 3 week per cycle). Then S‐1 monotherapy was continued for 8 cycles (Figure 1).

### 2.3 Endpoints

The primary endpoint was RP2D, which was defined as the dose immediately below the estimated MTD. The secondary endpoints were the 1‐year DFS rate and AEs. DFS was defined as the time from radical resection to recurrence of tumor or death, whichever occurred first. If the exact time could not be determined or the endpoint was not reached, the time of the last follow‐up or tumor assessment was recorded.

The study adopted the standard design of 3 + 3 dose escalation. When the number of participants in one dose level reached at least three and the evaluation data were obtained, the decision of dose escalation was based on investigators. If three participants at one dose level did not show a DLT, the next higher dose was carried out. If one participant had a DLT, the number of evaluable participants in this dose group was expanded to six; if there were no extra DLT occurring among the other three participants, the next dose level would be implemented; otherwise, this dose level would be defined as an intolerable dose. When reaching intolerable doses, the investigators had to decide whether to explore the appropriate dose or define this dose level as the MTD.

In this study, DLT was defined as any grade ≥3 non‐hematological toxicity according to NCI‐CTC AE 5.0, including peripheral neuropathy, nausea, vomiting, fatigue, and anorexia, that did not remit within 7 days after chemotherapy withdrawal and symptomatic treatment. Grade 3 of anemia, thrombocytopenia, or neutropenia sustained for >5 days, and/or febrile neutropenia (FN) during the treatment with the AS regimen for the first time were also defined as DLT. FN was defined as neutrophil <1.0 × 10^9^/L with a temperature of ≥38.3°C, or ≥ 38.0°C that lasted for >1 h.

### 2.4 Statistical analysis

Descriptive statistics are presented. The rate of DFS was calculated using the Kaplan–Meier survival method. Statistical analysis was performed with SPSS version 22.0 (IBM Corp.).

## 3 RESULTS

### 3.1 Characteristics of the participants

From July 2019 to June 2020, 12 participants with a median age of 48.5 years were enrolled in this study. The ECOG PS score was 0 in two participants (16.7%) and 1 in 10 participants (83.3%). There were five participants (41.7%) with stage IIIA disease, two participants (16.7%) with stage IIIB, and five participants (41.7%) with stage IIIC disease. The characteristics of the participants are listed in Table 1.

**TABLE 1:** Baseline characteristics of the participants

Characteristics	*n* = 12
Age, median (range)	48.5 (26–66)
Sex, *n* (%)	
Female	9 (75.0%)
Male	3 (25.0%)
ECOG PS score, *n* (%)	
0	2 (16.7%)
1	10 (83.3%)
Pathological stage	
IIIA	5 (41.7%)
IIIB	2 (16.7%)
IIIC	5 (41.7%)
Pathological T stage	
T3	5 (41.7%)
T4	7 (58.3%)
Pathological N stage	
N1	2 (16.7%)
N2	3 (25.0%)
N3	7 (58.3%)

Abbreviations: ECOG, Eastern Cooperative Oncology Group; PS, performance status.

### 3.2 Dose intensity

Participants received a total of 55 cycles of AS regimen, with a median cycle's number of 5 (range, 1–6). In the 260‐mg/m^2^ dose group, each participant was given 6 cycles of AS regimen. The rate of dose reduction was 32.7%, among which one participant in the 260‐mg/m^2^ group experienced dose reduction twice. The treatment status is presented in Tables 2 and 3.

**TABLE 2:** Treatment status of all participants

AS regimen	180 mg/m^2^ (*n* = 6)	220 mg/m^2^ (*n* = 3)	260 mg/m^2^ (*n* = 3)
Treatment cycles in total and in each participant	23 (3, 2, 6, 1, 6, 5)	14 (4, 5, 5)	18 (6, 6, 6)
Median treatment cycles at three levels	3.8	4.7	6
Dose reduction once, *n* (%)	10/23 (43.5%)	1/14 (7.1%)	4/18 (22.2%)
Dose reduction twice, *n* (%)	0	0	3/18 (16.7%)

**TABLE 3:** Reason for dose reduction or termination of chemotherapy

No. of patients	Different dose level (mg/m^2^)	Reason for termination of chemotherapy	Reason for dose reduction
1	180	Consent withdrawal	NA
2	180	Febrile neutropenia	Neutropenia
3	180	NA	Neutropenia
4	180	Subarachnoid hemorrhage	NA
5	180	NA	Neutropenia and leukopenia
6	180	Diarrhea	Diarrhea
7	220	Other reason^a^	Neutropenia and leukopenia
8	220	Neutropenia	NA
9	220	Intussusception	NA
10	260	NA	Peripheral neuropathy and liver injury
11	260	NA	Fatigue and dizziness
12	260	NA	Diarrhea, fever, and vomiting

Abbreviations: NA: not applicable.

a Due to COVID‐19 prevalence, the patient could not come to hospital to be treated on time.

### 3.3 MTD and RP2D

During the first cycle of AS regimen, one participant in the 180‐mg/m^2^ dose group experienced FN. Thus, three additional participants were enrolled in the 180‐mg/m^2^ dose group, and no severe AEs occurred. None of the participants in the 220‐ and 260‐mg/m^2^ dose groups showed DLT. Therefore, the MTD was not reached, and the RP2D of nab‐paclitaxel as adjuvant chemotherapy in AS regimen was 260 mg/m^2^ in DGC.

### 3.4 Treatment‐related adverse events

The commonly reported AEs included neutropenia (83.3%), leukopenia (66.7%), diarrhea (16.7%), and vomiting (16.7%). The most frequently observed grade 3–4 AEs were neutropenia (41.7%). The detailed AEs are listed in Table 4.

**TABLE 4:** Treatment‐related AEs

AE, *n* (%)	All (*n* = 12)		180 mg/m^2^ (*n* = 6)		220 mg/m^2^ (*n* = 3)		260 mg/m^2^ (*n* = 3)	
	Grade 1–2	Grade 3–4	Grade 1–2	Grade 3–4	Grade 1–2	Grade 3–4	Grade 1–2	Grade 3–4
Neutropenia	5 (41.7%)	5 (41.7%)	3 (25.0%)	3 (25.0%)	1 (8.3%)	2 (16.7%)	1 (8.3%)	0
Leukopenia	5 (41.7%)	3 (25.0%)	3 (25.0%)	2 (16.6%)	1 (8.3%)	1 (8.3%)	1 (8.3%)	0
Anemia	1 (8.3%)	0	0	0	0	0	1 (8.3%)	0
Febrile neutropenia	0	1 (8.3%)	0	0	0	1 (8.3%)	0	0
Peripheral neuropathy	0	1 (8.3%)	0	0	0	0	0	1 (8.3%)
Diarrhea	2 (16.7%)	1 (8.3%)	1 (8.3%)	1 (8.3%)	0	0	1 (8.3%)	0
Vomiting	2 (16.7%)	0	1 (8.3%)	0	0	0	1 (8.3%)	0
Fatigue	0	1 (8.3%)	0	0	0	0	0	1 (8.3%)
Dizziness	1 (8.3%)	0	0	0	0	0	1 (8.3%)	0
Rash	1 (8.3%)	0	1 (8.3%)	0	0	0	0	0
Alanine aminotransferase	1 (8.3%)	0	0	0	0	0	1 (8.3%)	0
Increased aspartate aminotransferase	1 (8.3%)	0	0	0	0	0	1 (8.3%)	0
Fever	1 (8.3%)	0	0	0	0	0	1 (8.3%)	0

Abbreviations: AEs, adverse events.

### 3.5 Survival

**FIGURE 2:**
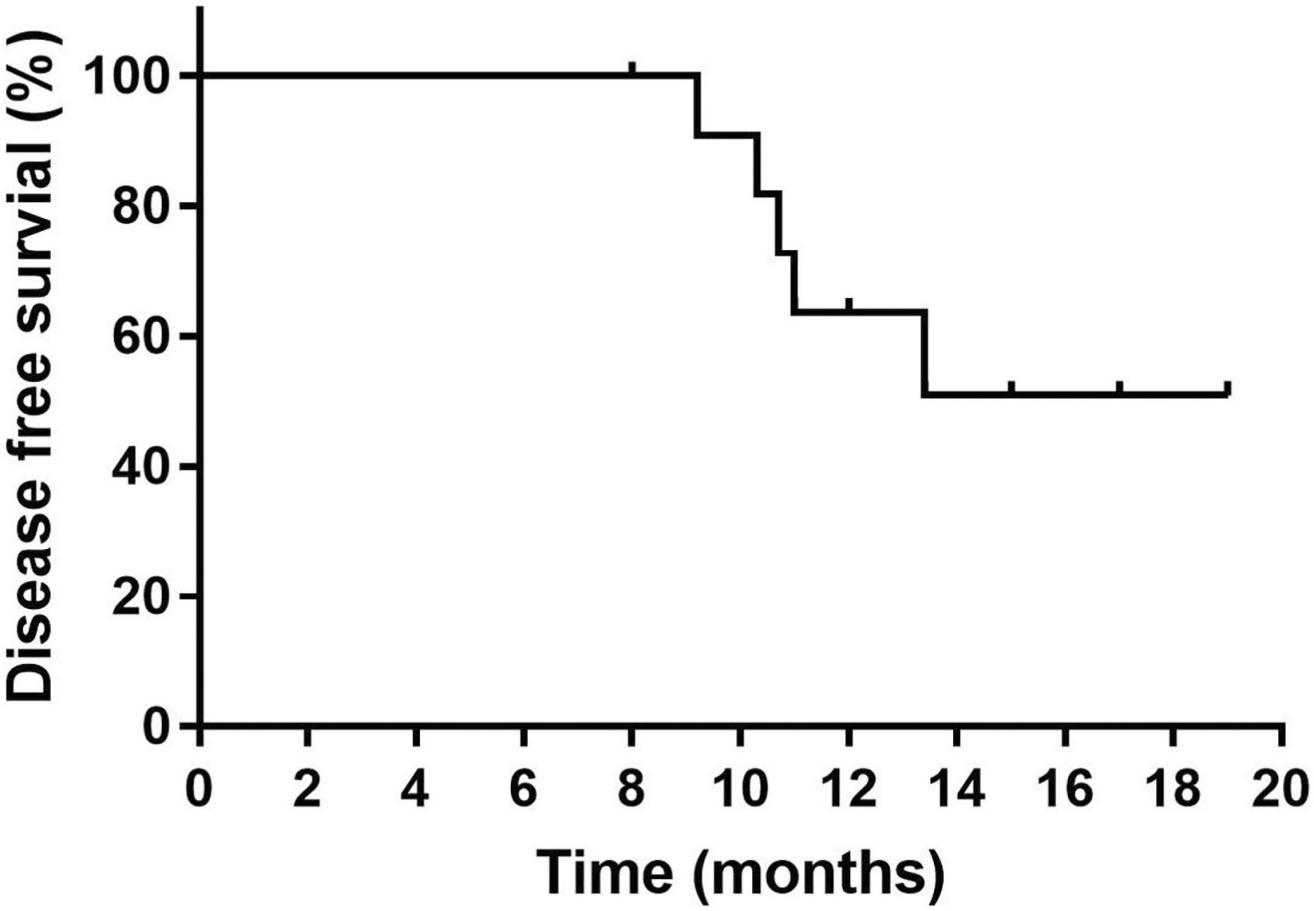
Disease‐free survival time by Kaplan–Meier analysis.

Up to January 2021, the median follow‐up time was 13.5 months (8.0–19.0 months). The participants in the 180‐ and 220‐mg/m^2^ dose groups were followed for >1 year. Five participants showed progressive disease before the last follow‐up (three in the 180 mg/m^2^ dose group and two in the 220 mg/m^2^ dose group). The median DFS was not reached (Figure 2). The 6‐, 12‐, and 18‐month DFS rates were 100%, 63.6%, and 50.9%, respectively.

## 4 DISCUSSION

The prognosis of DGC was worse than IGC, and S‐1 was confirmed to be effective in DGC. Furthermore, the optimal combinations of S‐1 have not been explored for patients with DGC. Thus, this study explored the MTD and the RP2D of the AS regimen as adjuvant treatment in stage III DGC. The results suggest that the RP2D of adjuvant nab‐paclitaxel was 260 mg/m^2^ in DGC. The AS regimen had a tolerable AE profile in stage III DGC.

Many researches have demonstrated the clinical benefits of postoperative adjuvant chemotherapy for stage II and III GC, while studies focusing on stage III DGC is still quite limited.^7^, ^9^ A phase III clinical trial (ACTS‐GC trial, *n* = 1059) evaluated the efficacy and safety of adjuvant chemotherapy with S‐1 following surgery versus surgery alone for stage II (excluding T1) or III GC.^10^ The 3‐year RFS rate in the S‐1 group and surgery only group was 72.2% and 59.6% (HR = 0.62; *p* < 0.001), respectively. Subgroup analysis revealed that the benefits were observed in stages II and IIIA, but not stage IIIB. Therefore, the results suggested that different regimens need to be explored for patients with GC, especially stage IIIB or IIIC disease. The CLASSIC^12^ study enrolled 1035 participants with stage II, IIIA, or IIIB GC to randomly receive capecitabine plus oxaliplatin (CAPOX) or surgery alone. The results showed that the 3‐year DFS in the two groups (HR = 0.56, *p* < 0.0001) was 74% and 59%, respectively. The 3‐year DFS for stage IIIA and IIIB diseases was 66% and 61%, respectively. Therefore, adjuvant chemotherapy with capecitabine and oxaliplatin has been recommended for GC after radical resection, especially for stage III GC.

The phase III study (JACCRO GC‐07, *n* = 1100) assessed the superiority of adjuvant S‐1 plus docetaxel to S‐1 monotherapy for stage III GC after D2 gastrectomy.^14^ And the interim analysis demonstrated that the rate of RFS in the S‐1 plus docetaxel group was higher than that in S‐1 monotherapy group (66% vs. 50%, HR = 0.632, *p* < 0.001). Therefore, the study was terminated early, when 915 participants were enrolled at that time. Although the rate of grade 3–4 AEs in the S‐1 plus docetaxel group was higher than that with S‐1 monotherapy, the AEs were well‐tolerated and manageable.

A phase II study compared the efficacy between S‐1 plus oxaliplatin (SOX) and CAPOX regimen as adjuvant chemotherapy for stage III GC. The 3‐year RFS rate in two groups was 70.9% and 67.8%, respectively.^27^ For participants with DGC who received SOX or CAPOX regimen, the 3‐year RFS rate was 71.8% and 57.1%, respectively. It seemed that regimens containing S‐1 might be a potential treatment option for DGC. A real‐world study found that IGC and DGC might be different in their sensitivity to chemotherapeutic agents, and the role of oxaliplatin as an adjuvant treatment among 580 participants was analyzed after D2 gastrectomy.^15^ The median DFS of DGC and IGC was 21.2 and 32.17 months, respectively (HR = 1.56, *p* < 0.001). The median OS for patients with DGC was 46.6 months, while the median OS for IGC has not yet reached (HR = 1.82, *p* < 0.001).

Therefore, patients with DGC have a poor prognosis,^15^ and more likely to develop peritoneal metastasis.^28^ A study in vivo suggested the synergistic effects of S‐1 and paclitaxel^26^ in breast cancer.^18^ Clinical studies also supported such synergistic effect, with ORRs of 50%–60% and good tolerability.^19^, ^20^, ^21^, ^29^ Compared with solvent‐based paclitaxel, nab‐paclitaxel was more effective and less toxic in the treatment of metastatic GC.^22^, ^23^, ^24^, ^25^ Similar results were confirmed in GC with peritoneal metastasis.^24^ Therefore, the adjuvant treatment regimen of nab‐paclitaxel plus S‐1 for stage III DGC deserved to be investigated.

The results showed that the dose level of nab‐paclitaxel could be tolerated up to 260 mg/m^2^ when combined with S‐1 based on the BSA. In the whole process of dose escalation, only one participant in the 180‐mg/m^2^ dose group displayed a DLT during the first cycle, which was FN, but DLT was not observed in the 220‐ and 260‐mg/m^2^ dose groups. It was consistent with a previously reported DLT of nab‐paclitaxel.^30^

In this study, the most frequent AEs in participants treated by nab‐paclitaxel combined with S‐1 were neutropenia (83.3%), leukopenia (66.7%), diarrhea (16.7%), and vomiting (16.7%). The most frequently observed grade 3–4 AEs was neutropenia, with an incidence of 41.7%. In ABSOLUTE study,^23^ nab‐paclitaxel was given as second‐line treatment for advanced GC. The incidence of neutropenia was 81%, of which grade 3–4 was 64%, and peripheral neuropathy was 85%, of which grade 3–4 was 20%. In ABSOLUTE study, nab‐paclitaxel (3 weekly and weekly) and paclitaxel (weekly) were used as single‐drug regimen. This study was combined with S‐1 which need rest time each cycle according to the protocol, so 3 weekly repeats would be more convenient. The safety of patients in this study was better than that in ABSOLUTE study, especially since the incidence of non‐hematological toxicity was lower.

Of note, all participants in the present study underwent surgery, but some patients with advanced GC are not candidates for surgical treatment. In such patients, the absorption of S‐1 might be different,^31^ leading to different AE profiles compared with operated patients. It will have to be further investigated in the future. Coincidentally, in this study the median age was 48.5 years, which was younger than that of general GC patients, might be due to DGC being more commonly observed in younger patients.^32^

This study had limitations. This was the phase I trial on a small number of patients. Only the MTD could be examined, and efficacy and safety will have to be tested in larger sample‐size patients of stage III DGC. The follow‐up time was relatively short, so the median DFS could not be observed.

In conclusion, the recommended dose of nab‐paclitaxel plus S‐1 as adjuvant therapy for patients with stage III DGC after D2 gastrectomy in the AS regimen can be up to 260 mg/m^2^ on day 1 of a 21‐day cycle. The MTD was not reached, and adjuvant chemotherapy with AS regimen in stage III DGC had a favorable toxicity profile.

## CONFLICT OF INTEREST

There was no competing interest was declared by all authors.

## AUTHORS' CONTRIBUTIONS

T.L. designed this study. Y.Y. contributed to the concept of this study design. Y.C., S.Y., W.L., Y.W. and Q.L. conducted this study. Y.C. drafted the article. The final version of the article was revised and approved by all authors.

## ETHICS APPROVAL AND CONSENT TO PARTICIPATE

The Ethics Committee of Zhongshan Hospital approved this trial (Approval number: B2019‐127R). The informed consent for study participation was obtained from all subjects.

## Trial registration

ClinicalTrials.gov (NCT03977220), June 6, 2019.

## Supporting information


**Appendix S1** Supporting information

## Data Availability

The data that support the findings of this study are available from the corresponding author upon reasonable request.
