# Supplementary material for: Phase I study of adjuvant chemotherapy with nab‐paclitaxel and S‐1 for stage III Lauren's diffuse‐type gastric cancer after D2 resection (NORDICA study)
Source: Cancer Med. 2022 Jun 25;12(2):1114–21. doi: 10.1002/cam4.4966 (PMC9883443; doi:10.1002/cam4.4966)
Supplement: Supplementary file 1 — Appendix S1 Supporting information [file CAM4-12-1114-s001.docx]

**Phase I study of nab-****paclitaxel combined with S-1 as adjuvant chemotherapy in stage III** **Lauren’s diffuse-type gastric cancer after D2 resection (NORDICA study)**

Inclusion criteria include: 1) Patients have good compliance and understand the research process of this study; 2) age 18-75 years old; 3) Histologically confirmed Lauren's diffuse-type of gastric or gastroesophageal junction adenocarcinoma; 4) Stage III according to AJCC/UICC Cancer Staging Manual (ed. 8); 5) Achieved D2 radical resection. 6) S-1 can be started within 4-6 weeks after operation, and it is well tolerated (no ≥3 grade treatment-related adverse events), then next cycle can be started as schedule; 7) The Eastern Cooperative Oncology Group Performance Status (ECOG PS) score: 0-1; 8) Bone marrow function: neutrophil absolute value (ANC)≥1.5×10^9^/L, platelet count≥100×10^9^/L, hemoglobin ≥90 g/L; 9) Liver and renal function: Serum creatinine≤1.0×upper limit of normal (ULN), aspartate transaminase (AST) and alanine transaminase (ALT)≤3.0×ULN, bilirubin total ≤1.5× ULN; 10) Postmenopausal or evidence of non-childbearing status. For women of childbearing potential should take a negative urine or serum pregnancy test. Patients with childbearing potential must agree to use effective contraception during the use of the study drug and within 6 months after last dose. 11) No contraindications for the use of Nab-paclitaxel and S-1; 12) Agree to provide histological samples.

Exclusion criteria include: 1) Patient had metastatic disease; 2) patients received neoadjuvant therapy or adjuvant therapy previously; 3) If the patient has a history of uncontrolled epilepsy, central nervous system disease or mental disorder, the researchers will judge whether it hinders the signing of informed consent or affects the patient’s oral medication compliance; 4) Clinically severe (that is, active) heart disease, such as symptomatic coronary heart disease, New York Heart Association (NYHA) classification of ≥ II (severe congestive heart failure) or severe arrhythmias requiring drug intervention, or a history of myocardial infarction within the last 12 months. Organ transplantation requires immunosuppressive therapy; 5) Patient has an ongoing or active infection, or other serious uncontrolled concomitant diseases. 6) Moderate or severe renal damage [creatinine clearance (Cr)≤ 50 ml/min (based on Cockroft and Gault equation)]; 7) Patients with dihydropyrimidine dehydrogenase (DPD) deficiency; 8) Patients has ≥ II grade peripheral neuropathy according to NCI-CTC AE v 5.0 criteria; 9) Patients with active HIV, HBV or HCV infection; 10) Patients with severe systemic diseases. 11) Patients with a known hypersensitivity to chemotherapeutic drugs or any excipients of nab-paclitaxel or S-1; 12) Postoperative complications that require clinical intervention and affect postoperative treatment, such as gastroparesis, dumping syndrome, and so on; 13) Other malignant tumors had occurred in the past 5 years, with the exception of cured cervical carcinoma in situ or non-melanoma skin cancer; 14) Participated in other trials within 30 days prior to the administration of the drugs containing in this trial.
